# Intriguing Developments in Cardiac Mapping, Defibrillation, and Pacing

**DOI:** 10.19102/icrm.2017.081208

**Published:** 2017-12-15

**Authors:** Claudio Tondo

**Affiliations:** ^1^Heart Rhythm Center at Monzino Cardiac Center, IRCCS, Department of Cardiac Sciences and Community Health, University of Milan, Milan, Italy

**Keywords:** Cardiac arrhythmia, cardiac mapping, cardiac pacing, defibrillator

As we are approaching the end of the current year, it is a good time to review the latest advancements of the technology in the field of clinical electrophysiology and cardiac pacing. I chose for inclusion in this commentary what I believe to be the most intriguing topics.

## Mapping

Mapping complex cardiac arrhythmias such as atrial fibrillation or different varieties of atrial flutter/atrial tachycardia is often challenging. In the last few years, high-density mapping has become of pivotal importance, as it enables us to identify the critical site(s) of any possible reentry circuits of these complex arrhythmias.

One of the latest revolutionary three-dimensional (3D) mapping systems is the AcQMap^™^ cardiac mapping system (Acutus Medical, Carlsbad, CA, USA). This system is based on the concept of analyzing dipole density rather than bipolar voltage during mapping. This difference is crucial, since the dipole density analysis considers the electrical activity across cell membranes and should provide much more detailed information in comparison with the bipolar voltage evaluation process, which takes into account a wider area of myocardial tissue and therefore might miss the core of electrical activation. The AcQMap^™^ system (Acutus Medical, Carlsbad, CA, USA) employs the use of a 25-mm basket catheter, which has 48 ultrasound transducers capable of collecting up to 144,000 ultrasound points per minute, and 48 engineered electrodes for the collection of 150,000 intracardiac unipolar samples per second. Electrodes with X, Y, and Z coordinate localization and ultrasound distances are combined to acquire the anatomy of the cardiac chamber, where the basket catheter is positioned; individual points are accumulated to form a surface with mesh-density equivalent to segmented computed tomography (CT), and post-processing of the surface data completes reconstruction.

The aim of the AcQMap^™^ system (Acutus Medical, Carlsbad, CA, USA) is to validate if complex arrhythmias, such as persistent atrial fibrillation, and atypical atrial flutters can be treated through the recognition of rotational activity in the atria and, therefore, the subsequent ablation of these rotational activities (which are almost stable over time) should promote the simplification of the underlying arrhythmia and, eventually, its termination. The preliminary data from the UN-COVER AF study are encouraging, with more expected in the year to come.

The electrophysiology community has shown an increasing interest in the so-called concept of “non-invasive” cardiac mapping with the use of vests that are worn, which provide high-density mapping through a high number of cutaneous electrodes (about 250). The goal is to identify the underlying mechanism(s) and the anatomic location(s) of varieties of cardiac arrhythmias prior to performing ablation. There are two systems available from Medtronic (Minneapolis, MN, USA) and EP Solutions (Marlton, NJ, USA), respectively. The recording of cardiac activity is matched with cardiac CT or magnetic resonance imaging findings to identify the anatomic location of the arrhythmia. This information is then used when the patient undergoes intracavitary 3D cardiac mapping to validate and confirm the analysis provided by the cutaneous electrocardiogram recording. The use of non-invasive mapping should allow for fast identification of the site of the arrhythmia’s mechanism and should shorten the conventional intracavitary mapping time and direct the operator toward the successful ablation site(s). If the use of these preliminary clinical data is confirmed as beneficial, the employment of noninvasive mapping may become more widespread in the field of cardiac arrhythmia diagnosis and treatment.

## Defibrillation therapy

Quite recently, it has been shown that the presence of long detection times in patients with single-chamber implantable cardioverter-defibrillators (ICDs) decreases mortality and unnecessary shocks. The supporting data are drawn from the ADVANCE III trial with the demonstration that prolonging the detection with antitachycardia pacing during charging is extremely safe and, thus, favors a significant and dramatic reduction of appropriate ICD therapies and reductions in the length and number of hospitalization stays. The findings of this trial are quite important with respect to their potential clinical impact and with regard to patients’ quality of life. In fact, with this ICD detection and intervention programming setup in place, the patient has no pain, no faintness, and an improved quality of life. Moreover, the study showed a drop of 30% in both general hospitalization and cardiovascular-related hospitalization rates and a decrease of 50% in mortality. Therefore, we can use the information from the ADVANCE III study and increase the VT detection times along with initiating the activation of other detection discriminators so as to decrease the number of appropriate shocks in selected patient groups without causing untoward clinical outcomes. We can rely upon the belief that these data can be of help to make ICD therapy much more physiologically acceptable to patients and thus favor its more frequent use in patients at increased risk of death.

Remaining in the field of pacing and defibrillation therapy, it is worth mentioning the novel implantable subcutaneous string defibrillator system (ISSD^™^; NewPace Ltd., Caesarea, Israel) under consideration use in for sudden cardiac death prevention. Instead of inserting the conventional active pulse generator (a can of about 60 cm^3^) over the ribs, the string subcutaneous defibrillator provides an active segment and two shocking coils, which are flexible structures to be positioned subcutaneously at the mid-sternum and lateral axillary line. The active segment contains all of the batteries, capacitors, and the communication system. The first 22 patients in Europe were enrolled in a trial at Homolce Hospital in Prague and the preliminary testing showed the majority of patients were successfully treated with 30 J or less, and some patients even with 20 J. The implantation time was very short (around 20 minutes), and one of the most promising findings is the high level of patient comfort. Additionally, the investigators expect to promote a final system with a lifespan of around 10 years and with a full recharge capacity for the defibrillator system.

## Pacing

We also owe special attention to the Micra leadless pacemaker (Medtronic, Minneapolis, MN, USA), which continues to demonstrate a high implant success rate and a very low level of major complications, even in the hands of operators with no prior experience with implantation of the device. This constitutes a great achievement, because it reveals an example of the state-of-the-art being used in a real-life setting, outside of randomized clinical trials. Registry results from the follow-up to the Micra investigational device exemption (IDE) trial showed a 99.2% successful implantation rate and a major complication rate due to cardiac effusion or perforation of only 1.6%. Moreover, all safety points and objectives were met and there were no dislodgements or infections.

More importantly, however, we need to discuss the post-approval registry that was designed to assess the safety and performance of this leadless pacemaker in the real world. The registry showed a 99.6% success rate for implantation, which is high, especially considering that 87% of implanters were without prior experience with the Micra leadless pacemaker (Medtronic, Minneapolis, MN, USA). Interestingly, the major complications rate was 1.5%, which is less than that found in the IDE trial. The higher success rate of implantation and the reduced rate of complications reported are likely due to the higher septal placement of the device, which usually guarantees optimal biophysical parameters and a reduced chance of perforation and pericardial effusion. Needless to say, focusing on physician training has also greatly contributed to these very positive results. These real-world data are the corroboration of the previous IDE trial data, and they reinforce the positivity of leadless pacing systems as a forerunner for the future of pacing.

